# Trajectories of Physical Activity and Screen Media Use in Toddlers and Related Parenting Practices: Child and Mother Physical Activity Study (CAMPAS)

**DOI:** 10.21203/rs.3.rs-7302701/v1

**Published:** 2025-08-27

**Authors:** Soyang Kwon, Nidhi S Gopagani, Isabella R Zylka, Sarah B Welch, Bridget Armstrong

**Affiliations:** Northwestern University; Northwestern University; Northwestern University; Northwestern University; University of South Carolina

**Keywords:** Preschooler Physical Activity Parenting Practices (PPAPP) questionnaire, ActiGraph accelerometers, screen time, early childhood, longitudinal study

## Abstract

**Background:**

Physical activity (PA) and screen media use are key behaviors for healthy development in early childhood. This study aimed to investigate 12-month trajectories of PA and screen time among toddlers and examine their associations with parenting practices.

**Methods:**

The Child and Mother PA Study (CAMPAS) recruited toddler-mother dyads in the US Chicago area from 2022–2024. Assessments were conducted at approximately 12, 18, and 24 months of age (Waves 1–3). Toddlers’ PA was measured using hip-worn ActiGraph accelerometers and their screen time was mother-reported. The PA Parenting Practices for Preschoolers (PAPPP) questionnaire was used to assess PA-encouraging and screen time-limiting parenting practices. Mixed-effects models were fit to model trajectories of moderate and vigorous-intensity PA (MVPA) and screen time over age. Multivariable regression models examined the associations between toddlers’ MVPA and screen time and their parents’ parenting practice scores.

**Results:**

The CAMPAS included 139 toddler participants (74 females). At Waves 1–3, average age was 13.6 (SD=1.7), 19.9 (SD=1.6), and 26.9 (SD=2.1) months, respectively. Average MVPA was 74 (95%CI=70–79), 73 (95%CI=69–77), and 80 (95%CI=75–85) minutes/day, respectively, with no significant change over time (*p*=0.13). Average screen time was 27 (95%CI=21–33), 46 (95%CI=38–55), and 59 (95%CI=50–69) minutes/day, respectively, significantly increasing over time (*p*<0.01). Every one-point higher PA-encouraging parenting practice score was associated with 9 additional minutes of daily MVPA (95%CI=2–15). For every one-point higher screen time-limiting parenting practice score was associated with 45 fewer minutes of daily screen time (95%CI=38–52).

**Conclusion:**

During the second year of life, toddlers exhibited a substantial increase in screen time but minimal changes in MVPA. Parenting practices were associated with toddlers’ MVPA and screen time. Findings suggest that promoting parenting practices that encourage PA and limit screen time may be an effective intervention strategy to support healthy PA and screen use development during this critical developmental period.

## Introduction

Globally, nearly 500 million people are projected to develop heart disease, obesity, diabetes, or other noncommunicable diseases attributable to physical inactivity between 2020 and 2030.^1^ As physical inactivity tends to persist over time,^2^ establishing healthy physical activity (PA) habits early in life is crucial. However, many preschool-aged children in the United States (US) do not meet recommended levels of PA,^3–6^ prompting increased interest in understanding PA behaviors among infants and toddlers.^7–9^ Yet, limited data exist on how PA behavior develops during these early years.

In contrast, screen time, including time spent on television (TV), tablets, and other digital devices, has been increasing among young children worldwide.^10^ A growing body of evidence^11–20^ suggests that excessive screen time negatively affects children’s development, including psychological well-being, language development, and cognitive development. The American Academy of Pediatrics’ guidelines discourage screen use before age 18 months and recommend high-quality programing with parental supervision thereafter.^21^ Nevertheless, screen exposure among American 1-year-olds has been rising,^22–24^ partly driven by the widespread availability of mobile devices.^40^ Longitudinal studies^25–27^ from the US and Australia have shown that screen media exposure increases substantially during the second year of life, rising by approximately 30 minutes between 12 and 18 months and an additional 30 minutes by 24 months. These findings suggest that age 1 is a critical period for establishing screen use habits.

Parents play a central role in shaping young children’s PA and screen time habits. Understanding how parenting practices relate to toddlers’ PA and screen use is vital for informing evidence-based early childhood interventions. PA-encouraging parenting practices are consistently linked to increased PA among children.^28^ Specifically among toddlers, cross-section studies have shown that PA-encouraging parenting practices, such as going on a walk with the child, setting aside time for active play with the child, and providing outdoor toys, were positively associated with toddlers’ PA levels,^29^ while screen time-related parenting practices, such as setting screen time limits and restricting mealtime screen use, were inversely associated with toddlers’ screen time.^30,31^ However, the majority of existing evidence is cross-sectional, and prospective studies are needed to strengthen causal inference.

To address this gap, the present study aimed to quantify longitudinal changes in PA and screen time over a 12-month period among 1-year-old children and to examine how parenting practices influence the development of these child behaviors. The objectives of this study were to 1) investigate the trajectories of PA and screen time from 12 to 24 months of age and 2) examine associations between PA- and screen time-related parenting practices and toddlers’ PA and screen time.

## Methods

### Study Participants

We conducted an observational, longitudinal study —Child and Mother Physical Activity Study (CAMPAS). CAMPAS is an ongoing cohort study aiming to investigate PA development from ages 1 to 3 years.^32^ Eligibility criteria for child participants included being between 10 and 15 months of age at baseline, having no cerebral palsy or other medical conditions precluding physical movement, and residing in the Chicago metropolitan area. Mothers were eligible if they self-identified as the child’s mother, were 18 years or older, lived with the child at least 50% of the time, and spoke English or Spanish. Participants were recruited between August 2022 and March 2024 through flyers distributed to various community locations and emails sent to potentially eligible families identified from a healthcare system’s electronic patient database. A total of 139 child-mother dyads completed baseline assessments and participated in 6- and 12-month follow-up assessments (referred to as waves 1, 2, and 3). Assessments were conducted either in-person at public locations (e.g., child indoor playrooms) or remotely. Further details about CAMPAS can be found in our prior publications.^32,33^

### Outcomes

Physical activity. PA variables of interest included daily moderate- and vigorous-intensity PA (MVPA), daily total PA, and average acceleration. Children wore a wGT3X-BT accelerometer on their hip for 7 days at each of waves 1 through 3. We extracted data collected between 6 AM and 10 PM, removing night-time data.^34–36^ Non-wear time, defined as ≥ 20 consecutive zero counts, was excluded.^37–41^ A valid wear day required ≥ 480 wear minutes within the 16-hour window.^37,41,42^ We included participants with ≥ 4 valid wear days in analysis.^41^ Light intensity was defined as 25–417 vertical counts per 15 seconds and moderate or higher intensity was defined as > 417 vertical counts per 15 seconds.^39,43^ Daily minutes spent in light-intensity PA and MVPA (minutes/day) were calculated. Daily total PA (minutes/day) was calculated by summing daily minutes spent in light-intensity PA and MVPA.^38,39^ In addition, we calculated average acceleration (mg) using the Euclidian norm minus on gravitational unit (ENMO)^44^ during wear time between 6 AM and 10 PM in the GGIR R package.^45^ Multiple days of PA measures were averaged per wave.

Screen time. A screen time variable of interest was daily time spent watching media on a TV, a tablet or a smartphone. Children’s screen time was proxy-reported by mothers, using two questions in the mother survey at waves 1 through 3: 1) “on average, how many minutes per day did your child spent watching things on a TV (including TV, DVD/Blu-ray or videos on apps like YouTube or Netflix through the TV)?” and 2) “on average, how many minutes per day did your child spend watching videos on a tablet or smartphone?”^24,46^ The number of minutes from the two items were summed to calculate daily screen time (minutes/day).

### Exposures

The primary exposure variables were scores on the PA-encouraging and screen time-limiting parenting practice subscales of the Preschooler Physical Activity Parenting Practices (PPAPP) questionnaire,^47^ reported by mothers at waves 1 and 3, but not at wave 2. The PA-encouraging parenting scale included 16 items, such as: “How often do you… a) play active games with your child (such as playing ball or racing)?; b) take your child to the park?; c) go on a walk with your child?; and d) say positive things to motivate your child to be more active?” The screen time parenting scale included three items: “How often do you… a) allow your child to watch TV for two hours or longer per day?; b) allow your child to use a tablet or smartphone for one hour or longer a day?; and c) keep your child occupied by letting him/her watch TV or use a tablet or smartphone?” Each item was rated in a 5-point Likert scale (1 = never; 2 = rarely; 3 = sometimes; 4 = often; 5 = always). Screen time items were reverse-coded so that higher scores indicated more frequent use of screen time-limiting practices. Scores from the PA-encouraging items were averaged to create a PA-encouraging parenting practice score. A screen time-limiting parenting practice score was created in the same manner.

### Covariates

Guided by a socioecological model for PA^48^ and an integrated model for young children’s screen use,^49^ this study considered covariates in multi-level contexts, including individual, interpersonal, and neighborhood factors influencing the development of PA and screen use behaviors. Based on the literature and our prior baseline data analysis,^29^ the following potential covariates were considered: sex, age, maternal employment (not employed vs. employed), presence of sibling(s) aged 0–5 years in the household (yes vs. no), neighborhood resources, weight-for-length percentile category, and gross motor competency. Mothers completed an online demographic survey at baseline, reporting on child sex, age, racial/ethnic background as well as maternal education attainment, household members, and residential address (Supplementary File 1). Using the residential address, we identified the census tract-level Child Opportunity Index (COI), a composite index that captures neighborhood resources and conditions that matter for children’s healthy development at a census tract level.^50^ Consistent with our prior analysis,^29^ 5 Chicago metropolitan COI categories were combined into 3 categories: very low or low, moderate, and high or very high. The information about maternal employment, household, and address data were updated at wave 3.

To track child growth, mothers provided a copy of the child’s most recent pediatric visit summary that contained length and weight measurements and a date of measurement at each wave. Body mass index (BMI) percentile was calculated using World Health Organization (WHO) standard,^51^ which was then dichotomized into < 95 and ≥ 95th percentile.^52^ Gross motor development was assessed using mother-reported Ages and Stages Questionnaire (ASQ) gross motor subscale at each wave.^53^ Based on the cutoff score defined in the ASQ scoring guides,^53^ gross motor subscale score was dichotomized into higher (above the cutoff; the child’s development appears to be on schedule) and lower growth motor competency (close to the cutoff [the child needs learning activities and monitoring] or below the cutoff [the child may need further assessments with a professional]).

### Statistical Analysis

All statistical analyses were conducted using SAS 9.4 (Cary, NC). Descriptive analyses were performed for all study variables. Loss to follow-up was evaluated by comparing characteristics of participants retained vs. lost to follow-up, using Chi-square tests and t-tests.

To model the trajectories of MVPA, total PA, and screen time over age, we used mixed-effects models. We did not consider the effect of walking ability in this analysis, as our prior analysis showed no differences in MVPA among participants who were able to independently walk (n = 83) and who were not (n = 54) at baseline^29^ and all participants were able to walk independently at wave 2. All available data were included to estimate coefficients using a maximum likelihood method under a missing-at-random assumption. Quadratic and linear polynomial terms for age were tested to capture potential non-linearity. Within-subject covariance was accounted for. The best-fitting model was selected based on Akaike Information Criterion (AIC) and Bayesian Information Criterion (BIC).

To examine associations between MVPA and parenting practices, a multivariable mixed-effects model was fit to predict MVPA by the PA-encouraging parenting practice score variable and covariates using waves 1 and 3 data. Wave 2 data were excluded because parenting practices were not assessed at wave 2. We selected covariates based on bivariate analyses between MVPA and potential covariates. Sex and growth interaction (i.e., sex and BMI category) was also tested, as prior studies reported a sex-BMI category interaction.^54,55^ We also explored whether a change in parenting practice scores was associated with a change in MVPA from wave 1 to wave 3 using a multivariable linear regression model. This linear regression model was additionally adjusted for baseline MVPA. These analyses were repeated for total PA and screen time outcomes.

## Results

The CAMPAS included 139 toddler participants (74 females). In the sample, 87.8% had mothers with college degree or higher education (Table 1). At wave 1, 70.5% had in-person visits, 54.8% at wave 2, and 30.5% at wave 3. Four participants (2.9%) did not complete the wave 2 assessment, and 21 participants (15.1%) did not complete the wave 3 assessment. Those who were retained (n = 118) had higher maternal education than those who were lost to follow-up (n = 21; *p* < 0.05). Other sociodemographic characteristics were not statistically significantly different.

The average age of participants was 13.6 (SD = 1.7), 19.9 (SD = 1.6), and 26.9 months (SD = 2.1) at waves 1, 2, and 3, respectively (Table 2). Average MVPA was 74 (95% CI = 70–79), 73 (95% CI = 69–77), and 80 (95% CI = 75–85) minutes/day at each of the waves. The proportion of children with zero screen time was 34.5% at wave 1, 12.6%, at wave 2, and 8.5% at wave 3. Average screen time was 27 (95% CI = 21–33), 46 (95% CI = 38–55), and 59 (95% CI = 50–69) minutes/day at each of the waves, respectively.

**Figure 1:**
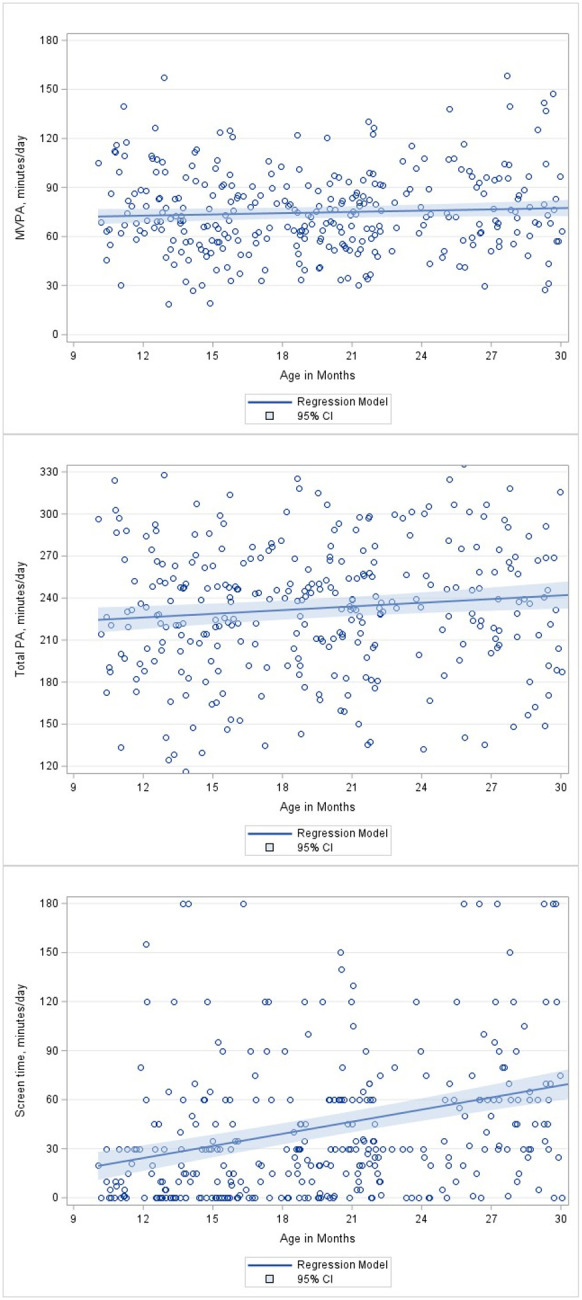
Scatter plots and modeled trajectories of moderate- and vigorous-intensity physical activity (MVPA), total physical activity (PA), and screen time.

PA and screen time trajectory models. Unadjusted mixed-effects models showed no significant change in MVPA over time (p = 0.13; Fig. 1a), while total PA increased by 10 minutes for 12 months (*p* < 0.01; Fig. 1b). Screen time was estimated as 24 minutes at 12 months and 54 minutes at 24 months, increasing by 30 minutes over the 12 months (*p* < 0.01; Fig. 1c).

Associations between parenting practices and MVPA and screen time. Multivariable mixed-effects models showed that every one-point higher PA-encouraging parenting practice score was associated with 9 additional minutes/day of MVPA (95% CI = 2, 15; Table 3). Every one-point higher screen time-limiting parenting practice score was associated with 45 fewer minutes/day of screen time (95% CI = 38, 52).

Multivariable linear regression models predicting changes from wave 1 to 3 found no significant association between changes in PA-encouraging parenting practice scores and changes in MVPA or total PA (Table 4). In contrast, a one-point increase in a screen time-limiting parenting practice score from wave 1 to 3 was associated with a 30-minute/day reduction in screen time during the same period (95% CI = 18, 42).

## Discussion

Summary of Findings. In this convenience sample of 139 1-year-olds living in urban and suburban areas of the US Midwest, we found that daily MVPA remained stable, while daily total PA increased by 10 minutes over a 12-month period. Screen time also increased by 30 minutes/day. Every one-point higher PA-encouraging parenting practice score was associated with 9 additional minutes of daily MVPA, and every one-point higher screen time-limiting parenting practice score was associated with 45 fewer minutes of daily screen time.

PA changes in Toddlerhood. This study is among the few to longitudinally examine sensor-based PA in toddlerhood. Our findings showed that toddlers maintained MVPA levels at 73–80 minutes on average over a 12-month period. These levels are notably higher than those reported in prior cross-sectional studies using the same MVPA definition: 47 minutes/day among an Australian sample of 1 year-olds^41^ and 50 minutes/day in a US sample of American 1 year-olds.^34^ They are also higher than a meta-analysis estimate (60 minutes/day) among children aged 1 and 2 year(s).^9^ Additionally, our findings showed that average daily total PA levels slightly increased from 227 to 241 minutes/day. These levels are similar to the 246 minutes found in the meta-analysis.^9^

We observed an increase in average acceleration from 12 to 24 months of age. Although the average acceleration metric, a key indicator of overall PA volume,^44,57^ facilitates comparison of PA across different accelerometer models,^57^ the absence of established reference values for toddlers limits its clinical interpretations. Future research is required to establish standardized PA metrics for young children.

Screen time changes in Toddlerhood. This study revealed an increase in screen time during the second year of life. The proportion of toddlers with no screen exposure declined markedly from 34.5% at baseline to 8.5% at one-year follow-up. A regression model estimated a 30-minute increase from 24 minutes at 12 months and 54 minutes at 24 months. This observed increase aligns with previous findings^25,26^ and confirm the early and accelerating adoption of screen media during the second year of life. However, the magnitude of the increase observed in this study is smaller than previously reported increases of approximately 60 minutes/day.^25–27^

This smaller increase in screen time could be partially explained by the sociodemographic characteristics of our sample, which predominantly included mothers with college degrees or higher education. Prior research has shown that children of mothers with lower educational attainment tend to have higher screen time.^26^ As a result, our findings reflect a higher-SES sample and may not fully be generalized to families facing greater socioeconomic disadvantage. The lower screen use could also be explained by variation in screen time assessment methods. For example, the Language in Little Ones (LiLO) Study^27^ utilized a Language Environment Analysis (LENA) technology to objectively capture exposure to electronic noise, including background TV exposure in an Australian sample in 2018–2021 and reported 88 minutes/day of screen time at age 12 months and 147 minutes/day at age 24 months. The Upstate KIDS Study^25^ relied on maternal report and found 53 minutes/day of screen time at 12 months and 115 minutes/day at 24 months in 2009–2013. While new technologies are making objective screen time measurement feasible,^60–62^ comprehensive assessment of children’s screen use remains complex, costly and, thus still relatively uncommon. Future research should prioritize the use of objective measures when possible, to better understand how screen use relates to child development.

PA and screen time parenting practices. We found that PA-encouraging parenting practices (e.g., verbal encouragement and logistic support) were associated with toddlers’ higher MVPA. This positive association is consistent with prior studies in toddlers^30^ as well as preschool-aged children.^63^ However, we found no association between changes in PA-encouraging parenting practices and corresponding changes in child PA over a 12-month period. This could be due, in part, to minimal variation in both PA-encouraging parenting practices and PA levels over time in our sample.

We found that screen time-limiting parenting practices were inversely associated with toddlers’ screen time. Specifically, each one-point higher screen time-limiting parenting practice score was associated with 45 fewer minutes of daily screen time. Longitudinally, when parents reduced the frequency of screen time-limiting parenting practices by one point (e.g., shifting from “often” to “sometimes”) over one year, their toddlers’ daily screen time increased by 30 minutes. This effect size is notable, especially considering the relatively low baseline screen exposure at age 1. These findings are consistent with prior study findings in toddlers^30^ as well as preschool-aged children.^63^ Parental screen time limits have been suggested as an effective strategy for reducing screen time among children.^64,65^ The current study underscores that even modest improvements in screen time-limiting practices, such as shifting from “sometimes” to “often,” could meaningful impacts on healthy screen media habits during this critical developmental period.

Limitations. Toddler’s screen time assessment relied on a maternal report, which may be subject to measurement error. Second, the sample consisted of relatively active toddlers, potentially limiting the generalizability of the findings to populations with lower PA. Third, the sample had relatively high maternal education attainment, which may affect the representativeness of the findings. Finally, the possibility of bias due to unmeasured confounding variables cannot be ruled out.

## Conclusions

During the second year of life, toddlers exhibited a substantial increase in screen time but minimal changes in MVPA. Parenting practices were associated with toddlers’ MVPA and screen time. Findings suggest that promoting parenting practices that encourage PA and limit screen time may be an effective intervention strategy to support healthy PA and screen use development during this critical developmental period.

## Supplementary Files

This is a list of supplementary files associated with this preprint. Click to download.

- SupplementaryFile1.docx

## Figures and Tables

**Table 1:** Participant characteristics at baseline

Variable	n (%)
Total	139 (100)
Sex	
Female	74 (53.2)
Male	65 (46.8)
Maternal education	
< 4-year college degree	17 (12.2)
≥ 4-year college degree	122 (87.8)
Maternal employment	
Employed	104 (74.8)
Not employed	35 (25.2)
Maternal marital status	
Married	113 (81.3)
Not married	26 (18.7)
Race and ethnicity	
Hispanic	29 (20.9)
Non-Hispanic Asian	5 (3.6)
Non-Hispanic Black	13 (9.3)
Non-Hispanic White	75 (54.0)
Non-Hispanic other	17 (12.2)
Child Opportunity Index	
Very low or low	33 (23.7)
Moderate	26 (18.7)
Hight or very high	80 (57.6)
Presence of a sibling aged 0–5 years	
Yes	68 (48.9)
No	71 (51.1)
Presence of a sibling aged 6–17 years	
Yes	28 (20.1)
No	111 (79.9)
Childcare attendance	
Yes	52 (37.4)
No	87 (62.6)
Body mass index category	
< 95th percentile	117 (84.9)
≥ 95th percentile	21 (15.1)
Gross motor competency	
Higher	110 (79.1)
Lower	29 (20.9)

**Table 2:** Physical activity, screen time, and related parenting practices by assessment timepoints

	Wave 1 (n = 139)	Wave 2 (n = 135)	Wave 3 (n = 118)
	mean ± SD	mean ± SD	mean ± SD
Age, months	13.6 ± 1.7	19.9 ± 1.6	26.9 ± 2.1
Accelerometer wear, days	6.8 ± 0.6	6.8 ± 0.6	6.7 ± 0.9
Accelerometer wear, hours/day	14.5 ± 1.7	14.6 ± 1.6	14.6 ± 1.7
MVPA, minutes/day	74 ± 26	73 ± 22	80 ± 26
LPA, minutes/day	152± 29	162 ± 28	162 ± 31
Total PA, minutes/day	227 ± 49	235 ± 44	242 ± 51
Average acceleration, m*g*	14.6 ± 4.5	18.6 ± 4.5	20.6 ± 5.4
Screen time	27 ± 38	46 ± 49	58 ± 13
PA-encouraging parenting practice score	3.7 ± 0.6	NA	3.8 ± 0.5
Screen time-limiting parenting score	4.5 ± 0.6	NA	4.1 ± 0.7

MVPA, moderate- and vigorous-intensity physical activity; NA, not applicable because parenting practice was not assessed at wave 2; PA, physical activity; SD, standard deviation.

**Table 3:** Multivariable mixed-effects growth models predicting MVPA, total PA and screen time, measured at waves 1 and 3.

	MVPA, minutes/day	Total PA, minutes/day	Screen time, minutes/day
	Coefficient (95% CI)	Coefficient (95% CI)	Coefficient (95% CI)
Intercept	37 (12, 60)	155 (108, 203)	211 (175, 247)
Age in years	1 (−3, 6)	8 (−1, 17)	11 (4, 19)
Sex			
Male	8 (0, 16)	18 (4, 32)	7 (−1, 16)
Female	Reference	Reference	Reference
BMI category			
≥ 95th percentile	−4 (−16, 8)	NA	NA
< 95th percentile	Reference	NA	NA
Male sex * ≥95th BMI percentile	12 (−6, 29)	NA	NA
Maternal employment			
Not employed	8 (−1, 16)	13 (−3, 29)	−5 (−15, 5)
Employed	Reference	Reference	Reference
Child Opportunity Index			
Very low or low	−8 (−17, 1)	−13 (−30, 5)	13 (2, 23)
Moderate	−5 (−15, 4)	−5 (−23, 13)	13 (2, 24)
High or very high	Reference	Reference	Reference
Presence of a sibling aged 0–5 years			
Yes	6 (−2, 13)	16 (2, 30)	NA
No	Reference	Reference	NA
Gross motor competency			
Lower	−3 (−12, 5)	−15 (−32, 2)	NA
Higher	Reference	Reference	NA
PA-encouraging parenting practices score	9 (2, 15)	14 (2, 26)	NA
Screen time-limiting parenting practice score	NA	NA	−45 (−52, −38)

BMI, body mass index; CI, confidence interval; MVPA, moderate- and vigorous-intensity physical activity; NA, not applicable; PA, physical activity.

**Table 4:** Multivariable linear regression models to examine associations between changes in parenting practices from Wave 1 to 3 and changes in physical activity and screen time over the same period

	Change in MVPA, minutes/day	Change in total PA, minutes/day	Changes in screen time, minutes/day
	Coefficient (95% CI)	Coefficient (95% CI)	Coefficient (95% CI)
Intercept	42 (11, 73)	170 (97, 243)	−4 (−53, 45)
Change in age, years	2 (−19, 24)	−16 (−60, 29)	18 (−22, 58)
Sex			
Male	6 (−2, 14)	−1 (−18, 16)	14 (−0.4, 28)
Female	Reference	Reference	Reference
BMI category			
≥ 95th percentile	1 (−10, 13)	−4 (−27, 19)	NA
< 95th percentile	Reference	Reference	NA
Maternal employment			NA
Not employed	−0.2 (−9, 9)	−0.4 (−19, 18)	−2 (−19, 15)
Employed	Reference	Reference	Reference
Child Opportunity Index			
Very low or low	3 (−7, 14)	6 (−16, 27)	26 (8, 44)
Moderate	−8 (−18, 3)	−15 (−36, 6)	3 (−16, 22)
High or very high	Reference	Reference	Reference
Presence of a sibling aged 0–5 years			
Yes	2 (−6, 10)	13 (−5, 30)	NA
No	Reference	Reference	NA
Gross motor competency			
Lower	−19 (−29, −9)	−33 (−54, −12)	NA
Higher	Reference	Reference	NA
Baseline PA or screen time^a^	−1 (−1, −0.4)	−1 (−1, −0.4)	−0.3 (−1, −0.1)
Change in PA-encouraging parenting practices score	6 (−5, 16)	14 (−7, 36)	NA
Change in Screen time-limiting parenting practice score	NA	NA	−30 (−42, −18)

BMI, body mass index; CI, confidence interval; MVPA, moderate- and vigorous-intensity physical activity; NA, not applicable; PA, physical activity.

a Baseline MVPA was used for a MVPA model, baseline total PA for a total PA model, and baseline screen time for a screen time model.

## Data Availability

Data may be available upon request to the corresponding author: Soyang Kwon, Soyang.kwon@northwestern.edu.

## Abbreviations

BMI: body mass index
CI: confidence interval
COI: Child Opportunity Index
MVPA: moderate- and vigorous-intensity physical activity
PA: physical activity
PAPPP: Physical Activity Parenting Practices for Preschoolers
